# Signals of demographic expansion in *Drosophila virilis*

**DOI:** 10.1186/1471-2148-8-59

**Published:** 2008-02-25

**Authors:** Patricia M Mirol, Jarkko Routtu, Anneli Hoikkala, Roger K Butlin

**Affiliations:** 1 School of Biology, The University of Leeds, Leeds LS2 9JT, UK; 2 Department of Biological and Environmental Science, P.O. Box 35, 40014 University of Jyväskylä, Finland; 3 Animal and Plant Sciences, The University of Sheffield, Sheffield S10 2TN, UK; 4 Museo Argentino de Ciencias Naturales, CONICET, Angel Gallardo 470, C1405DJR, Buenos Aires, Argentina

## Abstract

**Background:**

The pattern of genetic variation within and among populations of a species is strongly affected by its phylogeographic history. Analyses based on putatively neutral markers provide data from which past events, such as population expansions and colonizations, can be inferred. *Drosophila virilis *is a cosmopolitan species belonging to the virilis group, where divergence times between different phylads go back to the early Miocene. We analysed mitochondrial DNA sequence variation among 35 *Drosophila virilis *strains covering the species' range in order to detect demographic events that could be used to understand the present characteristics of the species, as well as its differences from other members of the group.

**Results:**

*Drosophila virilis *showed very low nucleotide diversity with haplotypes distributed in a star-like network, consistent with a recent world-wide exponential expansion possibly associated either with domestication or post-glacial colonization. All analyses point towards a rapid population expansion. Coalescence models support this interpretation. The central haplotype in the network, which could be interpreted as ancestral, is widely distributed and gives no information about the geographical origin of the population expansion. The species showed no geographic structure in the distribution of mitochondrial haplotypes, in contrast to results of a recent microsatellite-based analysis.

**Conclusion:**

The lack of geographic structure and the star-like topology depicted by the *D. virilis *haplotypes indicate a pattern of global demographic expansion, probably related to human movements, although this interpretation cannot be distinguished from a selective sweep in the mitochondrial DNA until nuclear sequence data become available. The particular behavioural traits of this species, including weak species-discrimination and intraspecific mate choice exercised by the females, can be understood from this perspective.

## Background

The development of methods to analyse intraspecific phylogenies has provided very valuable tools to understand how populations have been influenced by historical and contemporary processes [1,2]. Mitochondrial DNA has proved a very useful tool for reconstructing phylogenies of species and historical demographic events [3-8]. Its usefulness for the study of closely related taxa and populations within species lies in its very low rate of recombination, maternal inheritance, conserved structure, reduced effective population size and relatively high rates of evolution [6,7,9]. However, after an explosion of studies using mitochondrial DNA for phylogeographic inference, there is nowadays rising concern about making inferences based in this single molecule, due to problems related with recombination, effective population size, mutation rates, introgression and neutrality [10,11]. These problems influence the construction of species-level phylogenies most profoundly [e.g. [12]]. However, they may also confound estimations of demographic history and coalescence times within species. Nevertheless, if these drawbacks are taken into account when examining the evolutionary history of a taxon, mitochondrial DNA surveys can provide efficient means of detecting gene flow, levels of reproductive isolation, species boundaries and historical patterns of population structure [13].

The pattern of variation of mitochondrial DNA, permits inferences about a species' demographic history, including events such as population expansions and colonizations. The application of coalescent theory [14,15] for the analysis of population data has allowed inferences about past and present population size. A change in population size over time will be reflected in the DNA sequences in such a way that the analysis of the substitutions can indicate the direction and the timing of the change. It has been shown that demographic expansions lead to star-shaped genealogies [16], an excess of rare mutations [17] and a unimodal mismatch distribution [18]. Although spatial or range expansions also lead to an increase in the global effective size of a species, they lead to the same molecular signature as that of a purely demographic expansion if the migration rate between demes was large [19,20]. Otherwise, for relatively low levels of gene flow, multimodal mismatch distributions should be expected [19]. During the Pleistocene, global climate oscillations and the associated cycles of glaciation had a profound influence on the population distributions of many organisms [21-24]. Populations and species retracted their habitats to limited areas which served as refuges. Range expansions and colonizations followed by demographic expansions often happened after these events, when climate ameliorated, and it is possible to find the signals of those processes in contemporary populations.

The *Drosophila virilis *group comprises 13 species and subspecies divided into two clades, the virilis and montana phylads [25-30]. Throckmorton [25] suggested that the phylads diverged in the early Miocene, or not later than the Pliocene, when both of them entered the New World by way of Beringia. Divergence times between the phylads have been estimated to be from 7 Mya [31] to 11 Mya [29]. In general, species within the montana phylad have evolved more in terms of chromosomal re-arrangements, and are also more variable regarding the number of inversions segregating within populations than members of the virilis phylad [25], although species in this group show a higher number of fusions.

*Drosophila virilis *group species have been used as models in evolutionary studies for decades and their role in this kind of research will become even more important now that the whole genome of *D. virilis *has been sequenced http://insects.eugenes.org/species/data/dvir/. Within the virilis phylad, *Drosophila virilis *is a domestic species of nearly cosmopolitan distribution. The primitive karyotype is found in *D. virilis *and natural populations are exceptional in having no inversion polymorphism, in contrast to other species of the group. It has been proposed that *D. virilis *originated somewhere in the ancient deciduous forests of China or in arid regions such as Iran or Afghanistan, and remained isolated from the remaining species of the group until relatively recently [25]. Its ancestral role is supported by its monomorphic karyotype, an extremely high (about 50%) proportion of satellite DNA, and the distribution of various repetitive DNA elements [32].

*Drosophila virilis *also differs from other species of the group by the weak species-discrimination and intraspecific mate choice exercised by the females [e.g. [25,33]]. Although females are able to recognize species-specific characters of the male song, they do not require to hear the song before mating [34]. *D. virilis *strains originating from different parts of the species distribution have shown significant variation in male courtship song [33]. Rapid expansion into a novel habitat, not occupied by related species, may explain these characteristics. The ability of *D. virilis *to survive in various kinds of environments can partly be explained by its high thermotolerance [35] and high tolerance for ethanol [36]. Like several other cosmopolitan species, e.g. *D. melanogaster*, *D. virilis *possesses intraspecific genetic differences in thermotolerance with an obvious adaptive significance to local thermal conditions [37], which may make it easier for the species to occupy new habitats.

In this paper, we report an analysis of the demographic history of *D. virilis *using mitochondrial DNA sequence data, in order to provide an historical framework for evolutionary studies on life history and behavioural traits of the species and comparisons with other species in the group, especially *D. montana*, the most divergent species within the virilis group.

## Results and Discussion

We examined a total of 35 lines covering the species range: 9 from Japan, 9 from Eastern Europe and Central Asia, 5 from Western Europe, 2 from the United States and 10 from China (Table 1). These lines have been kept in captivity for widely varying numbers of years: since 1913 in the case of line 15010-1051.8 from California, and only since 2002 for the lines originating from wild flies collected in China.

**Table 1:** Lines of *D. virilis *used in the study, indicating, when it was available, year of collection and coordinates from which the line originates

*Drosophila virilis*	Line	Year	Coordinates
Matsuyama, Japan	A11	1973	
	A12	1973	
	B15	1973	
	B31	1973	
	B42	1973	
Sapporo, Japan	SBB	1986	43° 3'N, 141° 21'E
Sakata, Japan	SKT	1987	
Japan	Jap. Inv.		
Mishima, Japan	W158		
Hangzhow, China	15010-1051.47	1948	
Human, China	V-Hunan		
Zeziping, China	V-ZZP-01	2001	
Wuwei, China	V-WW-03	2002	
	V-WW-05	2002	
	V-WW-08	2002	
Lanzhou, China	V-EH-01	2002	
Dunghuang, China	V-DNH		
Nanjing, China	V-NANJING		
Qufu, China	V-QUFU		
Russia	15010-1051.52	1976	
Baku, Azerbaijan	1413	1974	40° 22'N, 49° 48'E
Jalta, Ukraine	1415	1973	46° 57'N, 37° 16'E
Batumi, Georgia	A		41° 39'N, 41° 39'E
Batumi, Georgia	S9	1970	41° 39'N, 41° 39'E
Yerevan, Armenia	1		40° 12'N, 44° 31'E
Mzheta, Caucasus	25		
Caucasus	1411	1973	45°N, 50°E
Tashkent, Uzbekistan	12		41° 18'N, 69° 16'E
Holland	W159		
Leeds, UK	LeedsA	1995	53° 47'N, 1° 32'W
	LeedsB	1995	
	1430	1981	53°N, 1°E
	1433	1982	53°N, 1°E
Pasadena, California, USA	15010-1051.0	1913	34° 8'N, 118° 8'W
Truckee, California, USA	15010-1051.8		39° 19'N, 120° 12'W

**Figure 1:**
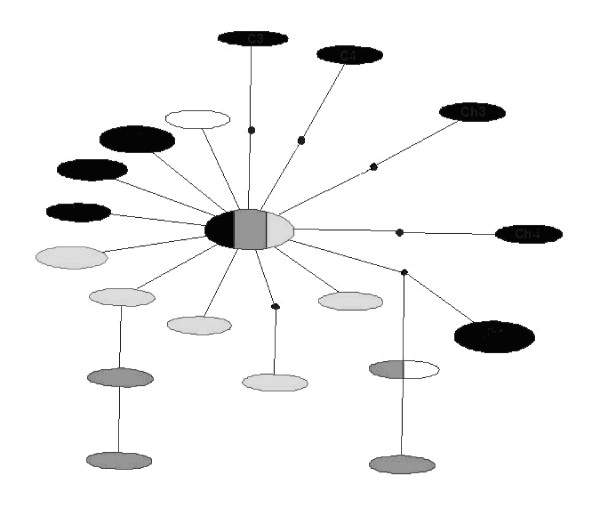
**Network obtained for the *D. virilis *haplotypes using statistical parsimony.** Haplotypes are represented by ellipses, the area of the ellipse is proportional to the frequency of the haplotype, points in the lines connecting circles indicate substitutions. Shading denotes region: white – North America, pale grey – Japan, dark grey – Western Europe, black – Asia and Eastern Europe.

The analysis was based on a total of 1358 base pairs of mitochondrial DNA sequence, 670 bp corresponding to the *cytochrome oxidase I *and 688 bp to the *cytochrome oxidase II *gene. There was a total of 19 haplotypes and, among them, 24 nucleotide substitutions, of which 20 were transitions and 4 transversions. Nucleotide diversity (per base) was 0.00185 ± 0.00114. The haplotype network (Figure 1), estimated using statistical parsimony, included one common haplotype, represented in six lines and present in all geographical regions except the USA. The second most frequent haplotype was found in four lines from China. The difference between these haplotypes was only two substitutions: in fact, the maximum number of differences between any pair of haplotypes was only 7 substitutions. There was no apparent geographical structure in the relationships among the haplotypes: the network was star-like with low levels of sequence divergence and a high frequency of unique mutations, indicating either a rapid population expansion or that selection has caused the rapid spread of a mitochondrial lineage carrying beneficial mutations. The limited number of lines sampled per region means that rare haplotypes specific to individual regions may have been missed. However, such haplotypes would contribute little to overall measures of differentiation.

When analysing demographic scenarios, where migration between populations, recombination and sexual reproduction usually play an important role, population history might not be tree-like. In these cases intraspecific genealogies constitute the appropriate way to make evolutionary inferences. The genealogical approach, which estimates parameters of the genealogical process, provides a coherent framework in which to consider recombination, migration, selection and other processes [38]. This approach takes into account several characteristics of the populations which make them different to species-level analysis, such as low divergence, ancestral nodes extant in the sampled population, multifurcations, reticulation and large sample sizes [1,2].

**Figure 2:**
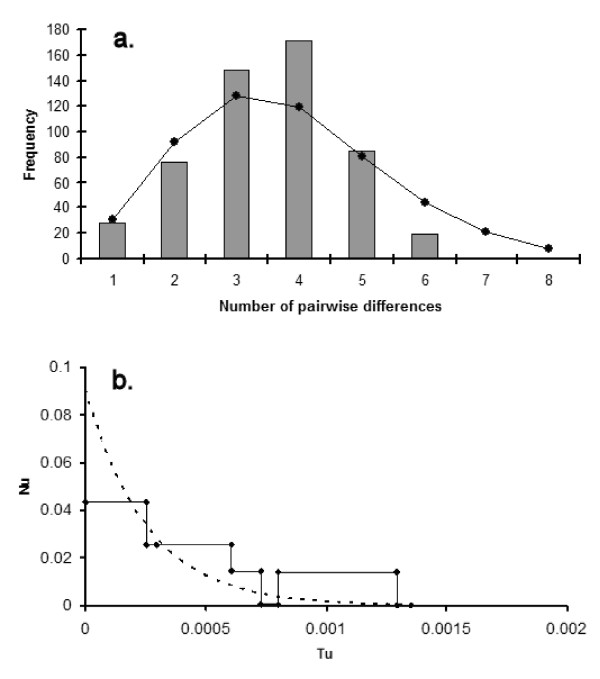
**Mismatch distribution (a) and generalised skyline plot (b) among haplotypes of *D. virilis*.** (a): Expectations from the stepwise growth model, fitted in ARLEQUIN, are superimposed. (b): Observed values (solid line) and fitted values from the best model (broken line) – see Table 2. Smoothing parameter (epsilon) was 4E-5 (maximum likelihood value from option 'maxepsilon' in GENIE). Nu: number of females, Tu: generations.

The mismatch distribution was smooth and unimodal (Figure 2a), but it departed from the expectations of the stepwise population expansion model (Harpending's raggedness index = 0.0721, p = 0.11, fit to the stepwise growth model, SSD = 0.0157, p = 0.03). There was an excess of unique mutations compared with that expected for a constant-size population (Table 2). We used the program FLUCTUATE to fit a model of exponential population expansion and estimate *θ*_0_, the present-day scaled population size (Table 2). The site categories and the rate of change were obtained using a maximum likelihood heuristic search in PAUP, using HKY85 as preferred model, following MODELTEST, with 5 rate categories, transition/transversion ratio of 2.3 and shape parameter of 0.105. Model comparisons using GENIE confirm that the exponential growth model was a good fit to the data and this can be seen in the skyline plot (Figure 2b) which indicates a rapid and recent expansion (Table 2).

**Table 2:** Summary statistics of mitochondrial variation and results from the FLUCTUATE and GENIE analyses.

						FLUCTUATE results		GENIE results		
n	H	θ_S_	θ_π_	Tajima's *D*	Fu's *F*	*Θ*_0 _(se)	*g *(se)	AICc Best model (second best model)	*Θ*_0 _(95% CI)	*g *(95% CI)
35	19	0.0044 (0.0015)	0.0019 (0.0011)	-2.0055 p < 0.01	-14.311 p < 0.0001	0.0499 (0.0103)	3532.6 (358.8)	Exp 149.83 (Log 148.38)	0.091 (0.027–0.410)	3951 (2610–5433)

Estimate (standard errors): n – number of lines surveyed, H – number of haplotypes, θ_S _– diversity (Waterson 1975), θ_π _– diversity (Tajima 1989), *θ*_0 _current estimate of 2Nμ, *g *– scaled population growth parameter. GENIE model abbreviations: Exp – exponential growth, Log – logistic growth.

The *Drosophila virilis *lines analysed in this study did not show any geographic structure among mitochondrial haplotypes. There was a very low number of substitutions differentiating haplotypes and they were commonly shared among locations. All analyses point towards a rapid population expansion over a short time-scale, which could be consistent with population growth following the end of the last glaciation and/or a shift into domestic environments. These are the most obvious possible causes of expansion, although they suggest population growth would have initiated less than 10,000 years ago which is more recent than the timing implied by the skyline plot, for example, which suggests continuous population growth for >50,000 years (using a substitution rate of 10^-8 ^per year). However, it is not possible to rule out alternative models, such as recent expansion from a smaller but more stable refugial population and time estimation should be treated with caution given the wide confidence interval on the expansion rate estimate and uncertainty about the substitution rate in *D. virilis*.

In natural populations, ancestral haplotypes may persist and be sampled together with their descendants [2]. The coalescence process predicts that high frequency haplotypes are likely to have been present in the population for a long time [2]. Rare haplotypes represent more recent mutations and are more likely to be related to common haplotypes than to other rare variants [39]. Therefore, ancestral haplotypes have greater probability of being central in a network, being common and having a broader geographic distribution [2]. Following these considerations, the central haplotype in the network appears to be ancestral (Figure 1), and because it is widely distributed gives no information on the geographic origin of the population expansion.

In their study on sequence variation in six X-linked genes of 21 *D. virilis *strains from different continents, Vieira and Charlesworth [40] found no fixed differences between the Asian strains and strains originating from Europe or North- or South-America, all the variants found outside Asia being also present in Asia, but not vice versa. These data were consistent with either a large population centred in Asia and a smaller migrant population elsewhere, or a large migrant population that went through a bottleneck [40]. Throckmorton [25] has previously suggested that *D. virilis *originated from an ancestral form in Asia, since the most primitive species of the *virilis-repleta *section of the genus *Drosophila *have been observed in Southeast Asia. This hypothesis recently received further support from a molecular phylogeny of the virilis section [41].

In a recent study, Huttunen *et al*. [42] analysed 48 microsatellite loci in 30 *D. virilis *strains, many of which were analysed here for mitochondrial DNA. Although a phylogenetic tree and STRUCTURE analysis showed only moderate clustering of the strains originating from Continental Asia, Europe, America and Japan, an assignment test using *a priori *information about the geographical origin of the strains gave high posterior probabilities for their correct assignment. Microsatellite variation also showed significant population differentiation, as measured by F_ST_, with evidence for isolation by distance. Variability detected by microsatellites can have a more recent origin than variation measured by mitochondrial DNA. Consequently, the lack of population differentiation in mitochondrial DNA could indicate shared ancestry while microsatellite variation between populations indicates that differentiation is in progress because current gene flow is restricted. Alternatively, the homogeneity of mitochondrial DNA could be the result of a recent selective sweep which did not disturb the pre-existing population structure for nuclear loci. Unfortunately, the use of laboratory strains prohibited testing for a demographic expansion using the microsatellite data.

There are several papers describing genetic variation in another species of the virilis group, *D. americana*, which, together with *D. lummei*, represents dispersal of the virilis group into the Neartic region [43-46]. *Drosophila americana *is represented by two chromosomal forms, *D. a. americana *and *D. a. texana*, differing by a fusion between the X and fourth chromosome and the frequency of chromosomal inversions. As in the case of *D. virilis*, phylogenetic analysis failed to resolve distinct clades between the chromosomal forms and also between geographic regions sampled, with F_st _values not significantly different from zero [43,44]. The absence of differentiation among the geographically distinct populations indicated either that gene flow homogenizes neutral sequence variation, even when different chromosomal rearrangements are involved, or that large populations retain shared ancestral polymorphisms [43]. Furthermore, and opposite to results obtained for *D. virilis *[42], an analysis of 27 microsatellite loci in 85 individuals from 6 different natural populations of *D. americana *showed no differentiation between populations [46], which confirms the existence of ongoing gene flow between populations of the species.

The pattern of geographic variation in haplotypes of *D. virilis*, and in *D. americana*, is in sharp contrast with the pattern recently described for another species of the group, *D. montana *[47]. In this case there was clear differentiation, based on both mitochondrial DNA and microsatellites, between lines from populations in Finland, Canada and USA. Both markers indicated the presence of at least two distinct populations, one in Eurasia and the other one representing the expansion of the species to the New World, with a divergence time between them estimated from 450,000 to 900,000 years ago, within the Pleistocene. Although *D. virilis *and *D. montana *belong to the same species group in the genus *Drosophila*, they represent different phylads and differ in many characteristics, including chromosomal variation, habitat preferences, and courtship behaviour. Differences in biogeographic history, reflected in the pattern of mitochondrial variation in the two species, could have been important in the origin of these characteristics and constitute the basis for the interpretation of their evolution.

## Conclusion

Mitochondrial DNA sequence variation in *Drosophila virilis *suggests a worldwide exponential population expansion during the Pleistocene, with extensive migration between demes. Slight differentiation at microsatellite markers results either from bottlenecking during or after expansion, or accumulation of differentiation across current barriers to gene exchange. The alternative possibility, that a selective sweep has homogenised mitochondrial sequences without disturbing pre-existing population structure for nuclear markers, cannot be excluded. However, a recent demographic expansion is consistent with the domestic habitat of *D. virilis *and helps to explain its lack of chromosomal polymorphism as well as evidence for a history of weak selection on mating behaviour by comparison with related species.

## Methods

### Drosophila stocks and sampling

In total, 35 *Drosophila virilis *strains, covering the species' range, were selected for analysis (Table 1). The stocks were collected during a time period covering almost 90 years, from 1913 to flies sampled in China in 2001/2002. A single individual from each strain, either from laboratory stocks or freshly caught, was used to extract DNA and for PCR amplification of the *COI *and *COII *mitochondrial genes.

### Amplification and sequencing of mitochondrial DNA

DNA was extracted from ethanol-preserved flies following a standard protocol [48], where the samples were homogenised in buffer and proteinase k, and DNA was extracted with chloroform-isoamyl alcohol and precipitated with isopropanol. The amplification of mitochondrial DNA was carried out with primers flanking the *COII *gene in the tRNA_LYS _and tRNA_LEU _[[49]; TL2: 5'-ATGGCAGATTAGTGCAATGG-3', TKN: 5'-GTTTAAGAGACCAGTACTTG-3'], which amplify an 850 bp fragment that includes the 688 bp *COII *gene, and COI-1460-F: 5'-ATCTATCGCCTAAACTTCAGCC-3' and COI-2195-R: 5'-ACTTCAGGGTGACCAAAAAATC-3' [50,51] which amplify the complete 670 bp corresponding to the *COI *gene. PCR reactions were performed in 50 μl volumes including 0.5 μM of each primer, 200 μM dNTPs, 1.5 mM MgCl_2 _and 1 U Taq polymerase (Bioline) in reaction buffer. Initial denaturation was for 7 minutes at 94°C followed by 35 cycles of 1 minute at 94°C, 1 minute at the annealing temperature (54°C for *COI *and 56°C for *COII*) and 1 minute at 72°C, and a final incubation of 5 minutes at 72°C. The products were purified using QIAquick columns (QIAGEN) and sequenced using the forward primer. No ambiguous sites were found in the sequences. Sequences (GenBank accession nos. DQ426800 to DQ426823) were aligned with CLUSTAL-V [52].

### Mitochondrial DNA analysis of population history and phylogeography

The partition homogeneity test, PHT [53], as implemented in PAUP 4.0, was used to test for incongruence between the *COI *and *COII *data sets. There was congruence between the data-sets and therefore the two fragments were combined for all subsequent analyses. ARLEQUIN 2.0 [54] was used to calculate pairwise distances between haplotypes, the mismatch distributions and tests of the standard neutral model for a demographically stable population (Tajima's *D *[55] and Fu's *F *[56]).

The program FLUCTUATE [57] was used to make simultaneous estimates of present day *θ *and the population growth rate *g*, assuming an exponential model of growth and using a maximum likelihood approach. The parameters used for the simulations were obtained by running a hierarchy of likelihood-ratio tests in Modeltest 3.0 [58] to choose the model of evolution with the best fit to the data. Skyline plots were constructed using GENIE v. 3.0 [59]. The starting trees were obtained using maximum likelihood with molecular clock enforced. The software requires that the genealogy is estimated under the assumption of a molecular clock [59]. GENIE was also used to calculate the fit to different models of population growth, with fit assessed using the corrected Akaike Information Criterion.

Networks of haplotypes were constructed based on statistical parsimony using the program TCS 1.06 [60].

## Authors' contributions

PMM carried out the molecular genetic studies, participated in the analyses and drafted the manuscript. JR participated in the molecular genetic studies. AH participated in the design of the study and helped to draft the manuscript. RKB participated in the design and in the analyses and helped to draft the manuscript. All authors read and approved the final manuscript.
